# Peel-and-Swiss roll biopsy: A practical technique for tissue sampling in fragile blistering skin conditions

**DOI:** 10.1016/j.jdin.2025.08.001

**Published:** 2025-08-18

**Authors:** Yu-Chen Jeng, Julia Yu-Yun Lee, Wei-Ting Liu

**Affiliations:** Department of Dermatology, National Cheng Kung University Hospital, College of Medicine, National Cheng Kung University, Tainan, Taiwan

**Keywords:** autoimmune blistering diseases, biopsy technique, exfoliative dermatoses, peeling, pemphigoid, pemphigus, severe cutaneous adverse reactions

## Challenge

Skin biopsy remains the gold standard for diagnosing exfoliative dermatoses, autoimmune blistering diseases, and severe cutaneous adverse reactions. However, in patients with widespread blistering or erosions, such as Staphylococcal scalded skin syndrome, Stevens-Johnson syndrome/toxic epidermal necrolysis, or pemphigus, conventional incisional or punch biopsies could be problematic. The procedures are traumatic, requiring local anesthesia, suturing, and posing infection risk; thus, they may be declined by patients or their families. In such cases, obtaining an adequate specimen for histopathologic evaluation without an invasive biopsy procedure presents a clinical challenge.

## Solution

**Fig 1:**
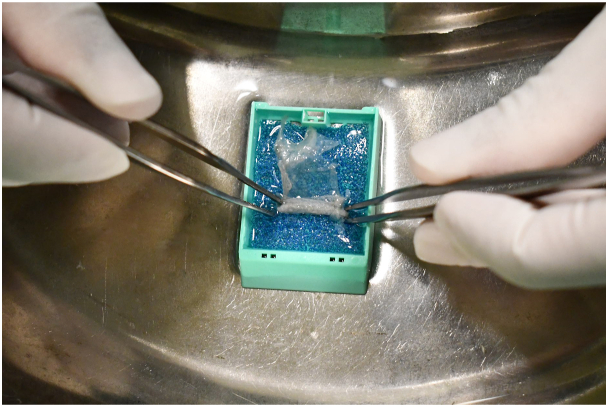
A freshly peeled off epidermal sheet cut off from the edge of a denuded skin is carefully rolled into a Swiss roll–like configuration to facilitate specimen fixation, embedding and cross-sectioning, and to maximize the histologic examination of the epidermal sheet in a nice vertical orientation.

**Fig 2:**
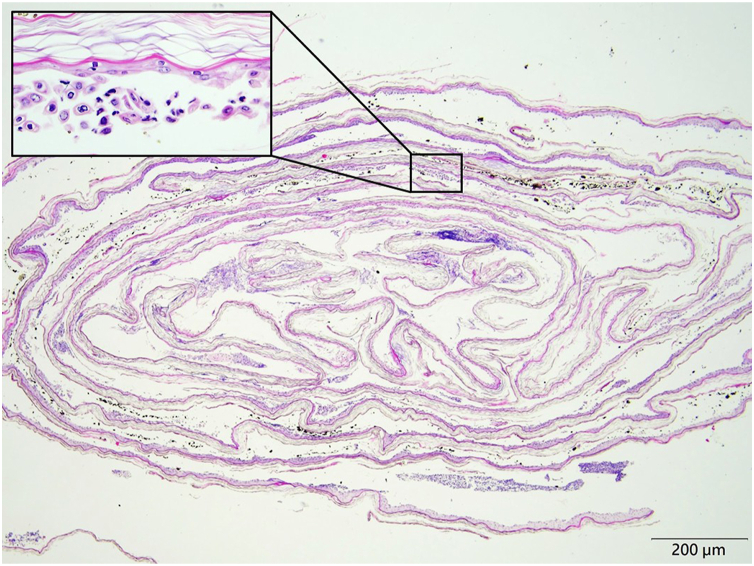
Section from a patient with pemphigus vulgaris showing the rolled-up epidermal sheet arranged in a scroll-like pattern, consistent with the Swiss roll configuration (hematoxylin and eosin, 40×). Acantholytic keratinocytes are identifiable on high-power view (figure inset).

For many years, we have been using a simple peel-and-Swiss roll skin biopsy technique as an adjunctive diagnostic method. From areas of freshly denuded skin, the detached epidermis is gently lifted using sterile forceps and cut off with scissors. The peeled-off specimen is soaked in normal saline to spread out evenly, then carefully rolled into a Swiss roll–like configuration (Fig 1). Rolling up the blister roof in this way can facilitate specimen embedding and cross-sectioning, thus maximizing the tissue available for histologic examination in vertical orientation (Fig 2, Video 1, available on www.jaad.org). This technique preserves sufficient epidermal architecture for diagnosis. In Staphylococcal scalded skin syndrome, detached basket-weaved stratum corneum with some neutrophils beneath is often seen (Supplementary Fig 1, available via Mendeley at http://doi.org/10.17632/4xcssh35wj.2). In pemphigus, acantholysis is readily identifiable (Supplementary Fig 2, available via Mendeley at http://doi.org/10.17632/4xcssh35wj.2).

A similar “jelly-roll” technique has been used in epidermolytic hyperkeratosis and linear immunoglobulin A bullous dermatosis.^1^^,^^2^ In that method, sloughed epidermis from the roof of a flaccid bulla was removed using a cotton-tipped applicator with back-and-forth motions. The sloughed skin was then wrapped around the cotton-tipped applicator for frozen or routine pathology sectioning. In contrast, our peel-and-Swiss roll approach is gentler and avoids the friction of cotton applicators, minimizing trauma to the blister roofs. The Swiss roll configuration also maintains tissue integrity during fixation and allows cross-sectioning of the roll to yield multiple well-oriented vertical sections of the epidermal sheet for microscopic examination.

While not replacing conventional biopsy methods, the peel-and-Swiss roll biopsy technique offers a simple, safe, nontraumatic alternative that yields diagnostic-quality tissue without increasing infection risk. Moreover, this technique would be easily acceptable to patients and their families, making it a practical adjunct in clinical dermatology.

## Conflicts of interest

None disclosed.

## Supplementary data

Video E1

## References

[1] Galler B., Bowen C., Arnold J., Kobayashi T., Dalton S.R. (2016). Use of the frozen section 'jelly-roll' technique to aid in the diagnosis of bullous congenital ichthyosiform erythroderma (epidermolytic hyperkeratosis). J Cutan Pathol.

[2] Khan S., Bae E., Mathieu R.J., Elco C., Massoud C.M., Firoz E.F. (2023). Revisiting the "jelly-roll" technique: new utility in diagnosing linear IgA bullous dermatosis mimicking toxic epidermal necrolysis. JAAD Case Rep.

